# Lattice atom-bridged chemical bond interface facilitates charge transfer for boosted photoelectric response

**DOI:** 10.1093/nsr/nwae465

**Published:** 2024-12-26

**Authors:** Mingwang Liu, Wenhong Yang, Runshi Xiao, Jinli Li, Rong Tan, Ying Qin, Yuxuan Bai, Lirong Zheng, Liuyong Hu, Wenling Gu, Chengzhou Zhu

**Affiliations:** State Key Laboratory of Green Pesticide, International Joint Research Center for Intelligent Biosensing Technology and Health, College of Chemistry, Central China Normal University, Wuhan 430079, China; State Key Laboratory of Green Pesticide, International Joint Research Center for Intelligent Biosensing Technology and Health, College of Chemistry, Central China Normal University, Wuhan 430079, China; State Key Laboratory of Green Pesticide, International Joint Research Center for Intelligent Biosensing Technology and Health, College of Chemistry, Central China Normal University, Wuhan 430079, China; Hubei Key Laboratory of Plasma Chemistry and Advanced Materials, Hubei Engineering Technology Research Center of Optoelectronic and New Energy Materials, Wuhan Institute of Technology, Wuhan 430205, China; State Key Laboratory of Green Pesticide, International Joint Research Center for Intelligent Biosensing Technology and Health, College of Chemistry, Central China Normal University, Wuhan 430079, China; State Key Laboratory of Green Pesticide, International Joint Research Center for Intelligent Biosensing Technology and Health, College of Chemistry, Central China Normal University, Wuhan 430079, China; State Key Laboratory of Green Pesticide, International Joint Research Center for Intelligent Biosensing Technology and Health, College of Chemistry, Central China Normal University, Wuhan 430079, China; State Key Laboratory of Green Pesticide, International Joint Research Center for Intelligent Biosensing Technology and Health, College of Chemistry, Central China Normal University, Wuhan 430079, China; Institute of High Energy Physics, Chinese Academy of Sciences, Beijing 100049, China; Hubei Key Laboratory of Plasma Chemistry and Advanced Materials, Hubei Engineering Technology Research Center of Optoelectronic and New Energy Materials, Wuhan Institute of Technology, Wuhan 430205, China; State Key Laboratory of Green Pesticide, International Joint Research Center for Intelligent Biosensing Technology and Health, College of Chemistry, Central China Normal University, Wuhan 430079, China; State Key Laboratory of Green Pesticide, International Joint Research Center for Intelligent Biosensing Technology and Health, College of Chemistry, Central China Normal University, Wuhan 430079, China

**Keywords:** photoelectrochemical sensing, atomic doping, *p-n* junction, interfacial chemical bonds, charge transfer

## Abstract

The construction of chemical bonds at heterojunction interfaces currently presents a promising avenue for enhancing photogenerated carrier interfacial transfer. However, the deliberate modulation of these interfacial chemical bonds remains a significant challenge. In this study, we successfully established a *p-n* junction composed of atomic-level Pt-doped CeO_2_ and 2D metalloporphyrins metal-organic framework nanosheets (Pt-CeO_2_/CuTCPP(Fe)), which enables the realization of photoelectric enhancement by regulating the interfacial Fe–O bond and optimizing the built-in electric field. Atomic-level Pt doping in CeO_2_ leads to an increased density of oxygen vacancies and lattice mutation, which induces a transition in interfacial Fe–O bonds from adsorbed oxygen (Fe–O_A_) to lattice oxygen (Fe–O_L_). This transition changes the interfacial charge flow pathway from Fe–O_A_–Ce to Fe–O_L_, effectively reducing the carrier transport distance along the atomic-level charge transport highway. This results in a 2.5-fold enhancement in photoelectric performance compared with the CeO_2_/CuTCPP(Fe). Furthermore, leveraging the peroxidase-like activity of the *p-n* junction, we employed this functional heterojunction interface to develop a photoelectrochemical immunoassay for the sensitive detection of prostate-specific antigens.

## Teaser

Atomic Pt doping-induced lattice oxygen-bridged bond reduces the carrier transport distance and optimizes the built-in electric field, thereby significantly improving the photoelectric performance.

## INTRODUCTION

Efficient interfacial separation and transfer of photogenerated charge carriers in heterojunctions play a pivotal role in determining the performance of sunlight-driven photoelectrochemical (PEC) catalytic and sensing applications [1–8]. The construction of a chemically bonded interface within heterojunctions has garnered significant attention due to its potential to create an atomic-level charge transport channel and reduce the energy barrier for charge transfer [9–11]. Despite the great success that has been achieved for the fabrication of various chemical bonds at the interface, the deliberate modulation of interfacial chemical bonds and the development of advanced functional interfaces remain challenging [12–14]. This limits our understanding of the relationship between bond structure and photoelectric performance to a certain extent.

Interfacial chemical bonds in heterojunction construction can typically be achieved through binding the surface functional groups of semiconductors. The surface-adsorbed or functionalized groups such as –OH, –NH_2_, –S, or organic molecules are often utilized as intermediates to build chemical bonds due to their convenience [15–20]. However, directly generating chemical bonds through binding lattice atoms of semiconductors can offer advantages such as shorter charge carrier transfer distances and faster charge transport channels [11,21]. Unfortunately, in many cases, the demanding reaction conditions associated with binding lattice atoms lead to the prevalence of adsorbed atoms, particularly in photoactive materials, undergoing surface modifications with functional groups. Tuning lattice atoms of semiconductors and optimizing the interfacial bonding mode will provide more opportunities for establishing efficient interfacial chemical bonds and an in-depth understanding of the transformation mechanism. Furthermore, the presence of large energy barriers for carrier migration at the adsorbed atom-induced chemical bond interface and the non-adjustability of the built-in electric field impose limitations on the performance of photoelectric systems [9,22–24]. Therefore, there is an urgent need to rationally design advanced heterojunction interfaces while simultaneously enhancing the interfacial driving force for the boosted photoelectric response.

**Scheme 1.**
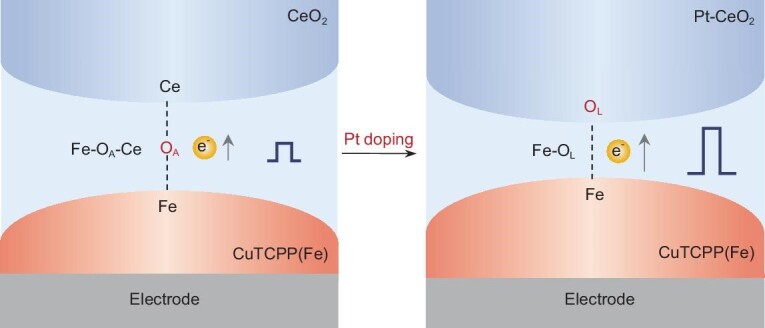
Schematic illustration of the mechanism of atomic-level Pt doping to optimize interface chemical bonding for enhanced PEC performance.

In this study, we present the fabrication of a *p-n* junction composed of atomic-level Pt-doped CeO_2_ and 2D metalloporphyrins metal-organic framework nanosheets (Pt-CeO_2_/CuTCPP(Fe)) using electrostatic coordination self-assembly, resulting in a notable 2.5-fold enhancement in photoelectric response compared with pristine CeO_2_/CuTCPP(Fe). Atomic-level Pt doping in CeO_2_ introduces an increase in oxygen vacancy density and lattice oxygen distortion, with a decrease in adsorbed oxygen, and theoretical computational modeling also shows that the introduction of Pt atoms leads to a protrusion of oxygen atoms in the surrounding lattice. These results facilitate the transition from indirect axial coordination bonding (Fe–O_A_) between Fe and adsorbed oxygen of CeO_2_ to direct axial coordination bonding (Fe–O_L_) with lattice oxygen (Scheme 1). Consequently, the Fe–O_A_–Ce with a bridge length of 4.41 Å is transformed to Fe–O_L_ with a length of 1.83 Å, thereby shortening the carrier transfer distance at the interface. Furthermore, the introduction of atomic Pt as an electron donor induces an overall upward shift in the band structure and Fermi level (*E*_F_) of CeO_2_. This shift narrows the difference between *p-n* junction bands and increases the difference of *E*_F_, resulting in less energy loss and more charge transfer during the interfacial carrier migration. *In-situ* Kelvin-probe force microscopy measurements conducted under dark conditions reveal a significantly higher surface potential for Pt-CeO_2_/CuTCPP(Fe) compared with CeO_2_/CuTCPP(Fe), providing evidence for increased charge separation and the generation of a stronger built-in electric field. The simultaneous optimization of interface migration distance and interface driving force greatly reduces the interface electron migration energy barrier from 22.9 to 2.1 eV. As a result, the photoelectric response exhibited a 2.5-fold improvement. In addition, coupling with the efficient enzyme-like catalytic activity of CuTCPP(Fe) and CeO_2_, we successfully fabricated a prostate-specific antigen (PSA) biosensing platform with a desirable analytical performance.

## RESULTS AND DISCUSSION

**Figure 1.**
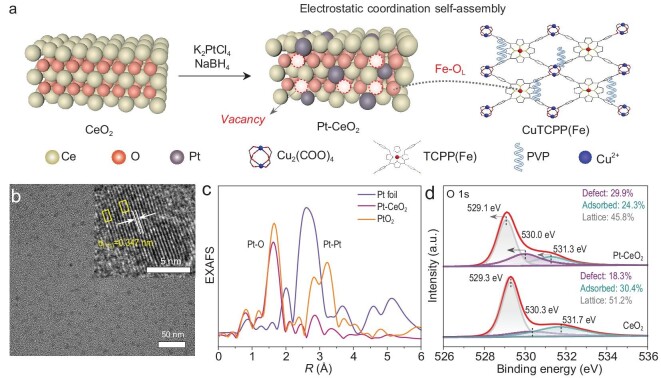
(a) Schematic illustration of the synthetic procedure for photoactive materials. (b) TEM (inset: HRTEM) image of Pt-CeO_2_. (c) FT-EXAFS spectra of Pt foil, Pt-CeO_2_ and PtO_2_. (d) High-resolution O 1s XPS spectra of Pt-CeO_2_ and CeO_2_.

The preparation route of the photoactive materials is illustrated in Fig. 1a. Atomic-level Pt was introduced in CeO_2_ by post-synthesis doping. Unless otherwise stated, Pt-doped CeO_2_ (Pt-CeO_2_) refers to Pt-CeO_2_ (5%) in the following. The content of the Pt atom in Pt-CeO_2_ was determined to be 1.6 wt% using inductively coupled plasma optical emission spectroscopy. The *p-n* junctions were fabricated by further electrostatic coordination self-assembly with CuTCPP(Fe). Transmission electron microscopy (TEM) and scanning electron microscopy were characterized to manifest the morphologies of CeO_2_, Pt-CeO_2_ and CuTCPP(Fe). CuTCPP(Fe) presents a nanosheet structure (Fig. S1), while Pt-CeO_2_ and CeO_2_ appear as nanoparticle morphology with average diameters of ∼6.8 and ∼6.5 nm, respectively (Fig. 1b and Fig. S2). As shown in the inset of Fig. 1b, a high-resolution TEM (HRTEM) image reveals that the lattice spacing in Pt-CeO_2_ is 0.347 nm, extending by ∼7.4% (0.024 nm) compared with 0.323 nm of CeO_2_, attributed to the doping of Pt into the CeO_2_ lattice. At the same time, obvious lattice distortion can be observed (yellow box in the inset of Fig. 1b), indicating that the introduction of Pt changes the crystal structure of CeO_2_.

X-ray absorption spectroscopy was employed to characterize the samples to further determine the chemical state and coordination environment of Pt. The X-ray absorption near-edge structure (XANES) of the Pt L_3_-edge for Pt-CeO_2_ and reference Pt foil and PtO_2_ are manifested in Fig. S3a. Comparing the absorption edge energy and white-line height of Pt-CeO_2_ with those of Pt foil, it is found that electrons flow from Pt to CeO_2_, indicating the cationic nature of Pt. The oxidation status of Pt species can be quantitatively assessed by integrating white-line peaks in XANES spectra (ΔXANES), and the average valence state of Pt is +3.7 (Fig. S3b and c) [25,26]. The Fourier transform of extended X-ray absorption fine structure (FT-EXAFS) spectra at the Pt L_3_-edge for Pt-CeO_2_ displays a distinct peak around 1.6 Å, corresponding to the Pt–O bond (Fig. 1c), which stands in stark contrast to the bond length observed in Pt foil (Pt–Pt, 2.6 Å). This observation confirms the atomic dispersion of Pt within Pt-CeO_2_. In addition, the absence of Pt crystalline was further demonstrated by X-ray diffraction analysis, providing evidence for the highly isolated Pt atomic dispersion in CeO_2_ (Fig. S4). The diffraction peak (111) in the inset demonstrates a slight displacement towards a lower angle edge, indicating that the introduction of Pt causes a lattice expansion of CeO_2_, consistent with the result of HRTEM. Meanwhile, X-ray photoelectron spectroscopy (XPS) analysis (Fig. S5a) was conducted to verify the chemical compositions. The introduction of Pt causes a shift of Ce 3d towards lower binding energy, indicating the donor characteristics of Pt doping and enabling electron transfer from Pt to Ce (Fig. S5b). As presented in Fig. 1d, the introduction of Pt leads to a large increase in defective oxygen and a decrease in adsorbed oxygen, indicating more serious lattice distortion. The signal intensity of Pt-CeO_2_ in variable-temperature electron paramagnetic resonance is significantly higher than that of CeO_2_, proving that doping of atomic Pt increases the surface oxygen vacancy concentration (Fig. S6). Thus, the lattice distortion caused by the increase of defects provides evidence for lattice oxygen mutation, making lattice oxygen more prone to axial coordination, while the reduction of adsorbed oxygen provides the possibility for more lattice oxygen coordination.

**Figure 2.**
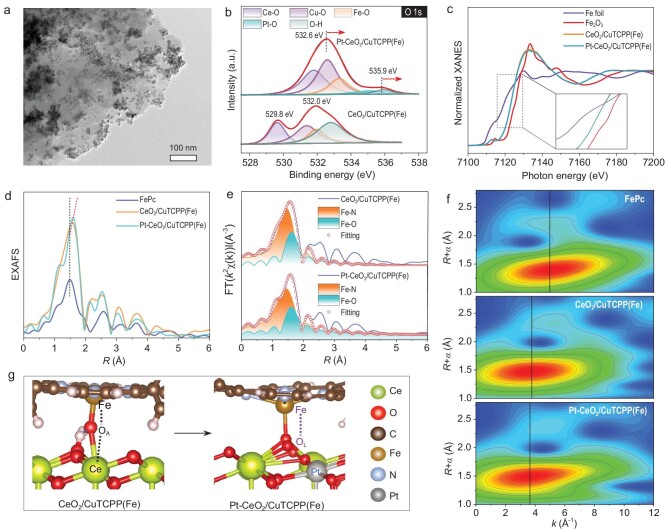
(a) TEM image of Pt-CeO_2_/CuTCPP(Fe). (b) High-resolution O 1s XPS spectra of CeO_2_/CuTCPP(Fe) and Pt-CeO_2_/CuTCPP(Fe). (c) Normalized Fe K-edge XANES spectra of Fe foil, Fe_2_O_3_, CeO_2_/CuTCPP(Fe) and Pt-CeO_2_/CuTCPP(Fe). (d) FT-EXAFS spectra at Fe K-edge of FePc, CeO_2_/CuTCPP(Fe) and Pt-CeO_2_/CuTCPP(Fe). (e) EXAFS fitting analysis. (f) WT-EXAFS contour plots at Fe K-edge of FePc, CeO_2_/CuTCPP(Fe) and Pt-CeO_2_/CuTCPP(Fe). (g) Schematic illustration of the interfacial Fe–O bond in different *p-n* junctions.

To prove the electrostatic coordination self-assembly of CuTCPP(Fe) and Pt-CeO_2_ nanoparticles, Zeta potential measurements were conducted. Different from the negatively charged CuTCPP(Fe), both CeO_2_ and Pt-CeO_2_ carry positive charges in pure water, indicating the feasibility of electrostatic self-assembly (Fig. S7). TEM and element distribution mapping images demonstrate the uniform dispersion of CeO_2_ and Pt-CeO_2_ on the CuTCPP(Fe) surface (Fig. 2a, Figs S8 and S9a). Lattice fringes of CeO_2_ and Pt-CeO_2_ are observed on HRTEM images of CeO_2_/CuTCPP(Fe) and Pt-CeO_2_/CuTCPP(Fe), respectively (Fig. S9b and c). To investigate the bonding patterns at the *p-n* junction interface, Fourier transform infrared, Raman, high-resolution XPS and XANES measurements were conducted. As illustrated in Fig. S10a, the bands at 463, 665, 721 and 879 cm^−1^ originate from the stretching vibration of Ce–O, Pt–O, Fe–O and Fe–N, respectively [27–29]. The doping of Pt induces a red shift in the Raman characteristic peak of CeO_2_ (Fig. S10b), verifying that Pt doping generates more oxygen vacancies [30]. In addition, the Raman spectra reveal the emergence of new characteristic peaks around 250 cm^−1^ assigned to the Fe–O bonds in CeO_2_/CuTCPP(Fe) and Pt-CeO_2_/CuTCPP(Fe), indicating the successful formation of the Fe–O bond as well (Fig. S10b). Detailed analysis of the high-resolution O 1s spectra of the *p-n* junctions shows a prominent Fe–O peak (Fig. 2b), indicating the formation of chemical bonds at the *p-n* junction interface through axial coordination. Compared with CeO_2_/CuTCPP(Fe), large offsets in the binding energy of O 1s indicate a stronger interfacial interaction in the Pt-CeO_2_/CuTCPP(Fe) *p-n* junction. The high-resolution XPS analysis of Fe 2p further verifies electron migration from CeO_2_ and Pt-CeO_2_ to CuTCPP(Fe) (Fig. S11). Thus, the introduction of Pt atoms strengthens the charge transfer from Pt-CeO_2_ to CuTCPP(Fe) at the *p-n* junction interface through the Fe–O chemical bond electron bridge, demonstrating that the Pt-CeO_2_/CuTCPP(Fe) interface has a stronger built-in electric field.

XANES and extended EXAFS measurements of Fe K-edge were used to further unravel local coordination geometry and electron states of the *p-n* junction. As shown in Fig. 2c, the absorption edge position of CeO_2_/CuTCPP(Fe) and Pt-CeO_2_/CuTCPP(Fe) was between that of Fe foil and Fe_2_O_3_, which suggests the oxidation state of Fe is between +2 and +3, consistent with the results of XPS. Furthermore, the fitted average oxidation state of Fe species for CeO_2_/CuTCPP(Fe) and Pt-CeO_2_/CuTCPP(Fe) were obtained from the XANES spectra, being +2.59 and +2.49, respectively (Fig. S12). The valence state of Fe in the *p-n* junction is smaller than that of Fe in the original CuTCPP(Fe), indicating that CuTCPP(Fe) is an electron acceptor in the assembled *p-n* junction, and a smaller valence state further verifies the stronger interfacial electron transfer of Pt-CeO_2_/CuTCPP(Fe), which is consistent with the above results. Due to the same Fe-N_4_ central structure, iron phthalocyanine (FePc) is used as a reference to further analyze the structural information of the *p-n* junction through the FT-EXAFS spectra of the Fe K-edge. As manifested in Fig. 2d, the FT-EXAFS spectra of FePc, CeO_2_/CuTCPP(Fe) and Pt-CeO_2_/CuTCPP(Fe) exhibit the main peak centered around 1.48, 1.57 and 1.60 Å in R-space, respectively, corresponding to the first shell coordinated atoms of isolated Fe atoms (N and O) [31,32]. The shift in position and the increase in the height of the main peak confirm that the coordination environment in the first coordination shell has changed. The larger shift reveals that Pt-CeO_2_ has stronger interaction and electron transfer with CuTCPP(Fe), indicating the significantly changed coordination environment. The least-squares EXAFS fitting of CeO_2_/CuTCPP(Fe) and Pt-CeO_2_/CuTCPP(Fe) were further performed to obtain quantitative structural parameters and the detailed coordination configuration (Fig. 2e and Table S1). As a result, the main peak centered is deconvoluted by Fe–N and Fe–O first shell coordination. The coordination numbers of the Fe–N and Fe–O in Pt-CeO_2_/CuTCPP(Fe) were determined to be 4.12 and 0.91 at distances of 2.03 and 1.81 Å, respectively. As a comparison, the coordination numbers of the Fe–N and Fe–O in CeO_2_/CuTCPP(Fe) were determined to be 3.85 and 0.82 at distances of 1.98 and 2.08 Å, respectively. Both CeO_2_/CuTCPP(Fe) and Pt-CeO_2_/CuTCPP(Fe) are the coordination structures of Fe-N_4_O_1_. The Fe–O bond of Pt-CeO_2_/CuTCPP(Fe) is shorter than that of CeO_2_/CuTCPP(Fe), which indicates that Pt atom doping has a greater influence on the central Fe-N_4_ structure due to the existence of oxygen vacancies and lattice mutation, achieving a transition from Fe–O_A_ to Fe–O_L_. Wavelet transform EXAFS (WT-EXAFS) was performed to further verify the coordination configuration of the Fe atoms (Fig. 2f). The WT-EXAFS contour plots of FePc, CeO_2_/CuTCPP(Fe) and Pt-CeO_2_/CuTCPP(Fe) vary in intensity maxima at 5.0, 3.8 and 3.6 Å^−1^, implying the Fe-N_4_ coordination environment can be further modulated by the axial Fe–O coordination at the interface. Based on the above experimental results, the local structure models of CeO_2_/CuTCPP(Fe) and Pt-CeO_2_/CuTCPP(Fe) are shown in Fig. 2g. It is indicated that the doping of Pt causes the transformation of the axial coordination mode from Fe–O_A_ to Fe–O_L_, leading to the change in the interfacial chemical bond bridge from Fe–O_A_–Ce to Fe–O_L_.

**Figure 3.**
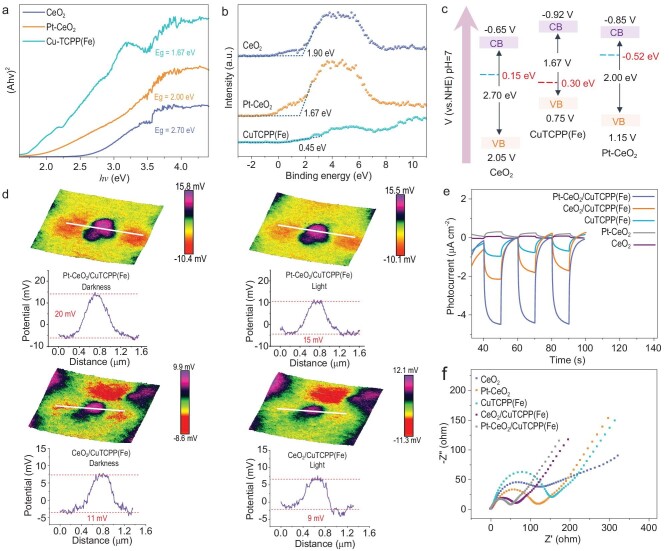
(a) Tauc plots of CeO_2_, Pt-CeO_2_ and CuTCPP(Fe) using (*F(R)hv*)^1/2^ (Kubelka–Munk parameter) as a function of the photon energy. (b) XPS valence band spectra and (c) schematic energy band diagram of CeO_2_, Pt-CeO_2_ and CuTCPP(Fe). (d) KPFM measurements for 3D surface potential distribution and corresponding line-scanning of CeO_2_/CuTCPP(Fe) and Pt-CeO_2_/CuTCPP(Fe) in darkness and under illumination. (e) Photocurrent response and (f) EIS spectra of photoactive materials.

The photoelectric response of different doping ratios was optimized, and the doping concentration of Pt was finally selected as 5% (Fig. S13). The influence of atomic Pt doping on the *p-n* junction was subsequently analyzed using band structure characterization and *in-situ* irradiated XPS. From the Tauc plots of ultraviolet-visible diffuse reflection spectral conversion, the bandgaps (*E*_g_) of CeO_2_, Pt-CeO_2_ and CuTCPP(Fe) are 2.70, 2.00 and 1.67 eV, respectively (Fig. 3a and Fig. S14). The positive slope of the Mott–Schottky curve tangent of CeO_2_ and Pt-CeO_2_ indicates their n-type characteristics, while CuTCPP(Fe) exhibits p-type behavior. The flat-band potentials (*E*_fb_) of CeO_2_, Pt-CeO_2_ and CuTCPP(Fe) are −0.55, −0.75 and 0.65 V (*vs*. Ag/AgCl, pH 7), respectively (Fig. S15). Therefore, the valence bands (VB) of CeO_2_, Pt-CeO_2_ and CuTCPP(Fe) can be calculated, being 2.05, 1.15 and 0.75 V (*vs*. normal hydrogen electrode (NHE), pH 7), respectively [33]. Accordingly, their conduction bands are −0.65, −0.85 and −0.92 V (*vs*. NHE, pH 7), respectively. In general, *E*_F_ is the energy difference between the XPS VB values (Fig. 3b) and VB [34]. In this case, the *E*_F_ of CeO_2_, Pt-CeO_2_ and CuTCPP(Fe) is calculated as 0.15, −0.52 and 0.30 eV (*vs*. NHE, pH 7), respectively. As illustrated in Fig. 3c, compared with CeO_2_, the energy band and *E*_F_ of Pt-CeO_2_ are smaller after doping with Pt. This disparity in *E*_F_ is conducive to electron transfer of the *p-n* junction in the dark state. The pathways for interfacial charge transfer were further investigated using *in-situ* irradiated XPS. Compared with CuTCPP(Fe), the binding energies of Cu 2p for the *p-n* junction negatively shift in darkness and positively shift under light irradiation, demonstrating the electron transfer from Pt-CeO_2_ and CeO_2_ to CuTCPP(Fe) in darkness, while opposite transfer occurs under illumination (Fig. S16a). Similarly, the binding energies of Ce 3d for the *p-n* junction exhibit an opposite shift compared with Cu 2p, further verifying the above carrier transfer direction at the interface (Fig. S16b). The corresponding schematics of the band structure and electron transfer in the PEC process are displayed in Fig. S17 respectively. The energy band difference between Pt-CeO_2_ and CuTCPP(Fe) is significantly smaller, indicating less loss of redox capacity. Furthermore, due to the high *E*_F_ of CeO_2_ and Pt-CeO_2_ in comparison with CuTCPP(Fe), the electrons from CeO_2_ and Pt-CeO_2_ will migrate spontaneously to CuTCPP(Fe) upon close contact to equalize their *E*_F_ levels. Consequently, a space-charge layer, formed by the migration of charges, will create a built-in electric field directed from CeO_2_ and Pt-CeO_2_ towards CuTCPP(Fe). Simultaneously, the bands of CeO_2_ and Pt-CeO_2_ bend upward, whereas that of CuTCPP(Fe) bends downward. In general, the *E*_F_ difference determines the strength of the built-in electric field. Pt atom doping increases the discrepancy in *E*_F_ between semiconductors, thus enhancing the built-in electric field for fast charge transfer.

Generally, the built-in electric field is closely related to the surface potential of the materials, which can be characterized by *in-situ* Kelvin-probe force microscopy (KPFM) [35]. When irradiated under light, KPFM provides direct visualization of the direction of photoinduced charge separation and transfer in semiconductors. As can be seen in Fig. 3d, the Pt-CeO_2_/CuTCPP(Fe) *p-n* junction exhibits a more pronounced three-dimensional (3D) surface potential of 20 mV than that of CeO_2_/CuTCPP(Fe) (11 mV) in darkness, indicating a stronger built-in electric field. Under light irradiation, the surface potential of Pt-CeO_2_/CuTCPP(Fe) and CeO_2_/CuTCPP(Fe) decreased by 5 and 2 mV in comparison with those in darkness, respectively, revealing the opposite direction of photoelectron migration forced by the built-in electric field. Consequently, atomic donor doping amplifies the built-in electric field of the *p-n* junction, thereby generating a powerful driving force for the accelerated separation and directed migration of carriers. Subsequently, the photoelectric performance of *p-n* junctions was investigated. As shown in Fig. 3e and Fig. S18, the optimized Pt-CeO_2_/CuTCPP(Fe) *p-n* junction exhibits a superior photocurrent response compared with the individual components and CeO_2_/CuTCPP(Fe), thus demonstrating the enhanced charge transfer capability of the Pt-CeO_2_/CuTCPP(Fe). The electrochemical impedance spectroscopy (EIS) Nyquist plots of the Pt-CeO_2_/CuTCPP(Fe) display a semicircle with the smallest radius, indicative of the lowest charge-transfer resistance (Fig. 3f). The quenching of steady-state photoluminescence (PL) can describe the suppression of intrinsic radiation recombination of photogenerated electron-hole pairs. It is found that the PL intensity of Pt-CeO_2_/CuTCPP(Fe) is lower than that of CeO_2_/CuTCPP(Fe), which proves that Pt-CeO_2_/CuTCPP(Fe) can effectively inhibit the carrier recombination (Fig. S19) [36].

**Figure 4.**
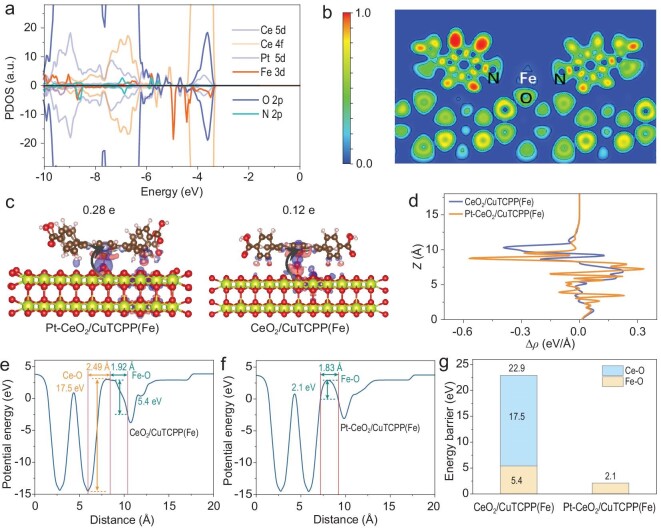
(a) PDOS for Pt-CeO_2_/CuTCPP(Fe). (b) 2D ELF of Pt-CeO_2_/CuTCPP(Fe). (c) Charge density difference for Pt-CeO_2_/CuTCPP(Fe) and CeO_2_/CuTCPP(Fe). (d) < Δρ(z) > averaged through the *xy* plane of the layers corresponding to different locations along the *z*-axis. The potential energy of CeO_2_/CuTCPP(Fe) (e) and Pt-CeO_2_/CuTCPP(Fe) (f). (g) Interfacial charge transfer energy barriers of CeO_2_/CuTCPP(Fe) and Pt-CeO_2_/CuTCPP(Fe).

To deeply understand the influence of different bonding modes on the built-in electric field and interfacial charge transfer of heterojunction, first-principle calculations based on density functional theory were carried out. The method and parameters of the theoretical calculations are given in the Supplementary data. The Pt-CeO_2_ model reveals the presence of bridging oxygen atom protrusions in the structure, which can serve as the lattice oxygen sites for adsorbing Fe (Fig. S20). Partial density of states (PDOS) and electronic location function (ELF) were employed to reveal the bonding interaction between Pt-CeO_2_ and CuTCPP(Fe). As shown in Fig. 4a, substantial overlap between Fe 2p, N 2p and O 2p orbitals is observed, indicating that Fe atoms in CuTCPP(Fe) can hybridize with O atoms in Pt-CeO_2_ to form N–Fe–O_L_ bonds [37]. In addition, the ELF reveals that N and O have strong interactions with Fe, which unambiguously unmasks that Pt-CeO_2_ and CuTCPP(Fe) can form N–Fe–O_L_ bonds at the atomic-level interface (Fig. 4b) [38,39]. Furthermore, the charge density difference of Pt-CeO_2_/CuTCPP(Fe) also reflects the identical interface charge distribution with ELF (Fig. 4c), where electron cloud density accumulates on CuTCPP(Fe), while depleting on O atoms of Pt-CeO_2_. Through Bader charge analysis, electron transfers of 0.28 and 0.12 e occur via the Fe–O_L_ and Fe–O_A_–Ce electronic bridges from Pt-CeO_2_ and CeO_2_ to CuTCPP(Fe), respectively. This indicates a stronger built-in electric field in the Pt-CeO_2_/CuTCPP(Fe). The contour plots of Δρ reveal that the charge redistribution region at chemical bond interfaces of Pt-CeO_2_/CuTCPP(Fe) is more pronounced and concentrated (Fig. 4d) [21,24], indicating that Pt-CeO_2_/CuTCPP(Fe) has a stronger built-in electric field as well. The potential energy and chemical bond length of the two models were calculated to gain an in-depth insight into the role of different interfacial environments in charge transfer. Given the carrier is transported between the two phases by the interfacial chemical bond of heterojunction, the interfacial chemical bond length determines the interfacial migration distance. For CeO_2_/CuTCPP(Fe), the energy barrier of 22.9 eV to be overcome for electron transfer from CeO_2_ to CuTCPP(Fe) is the sum of 5.4 eV of Fe–O_A_ and 17.5 eV of Ce–O_A_, and the electron transfer distance 4.41 Å at the interface is the sum of the bond lengths of Fe–O_A_ (1.92 Å) and Ce–O_A_ (2.49 Å) (Fig. 4e and g). Nevertheless, the energy barrier and transfer distance can be decreased to 2.1 eV and 1.83 Å by changing the bonding mode to the Fe–O_L_ bridge (Fig. 4f and g). The aforementioned findings unequivocally establish that lattice atom-bridged interfacial chemical bonding offers superior energetics and spatial characteristics for efficient electron transfer. Therefore, adjusting the mode of interfacial bonding facilitates a reduction in energy barriers and migration distances associated with charge transfer within heterojunctions, thereby significantly promoting interfacial charge flow.

**Figure 5.**
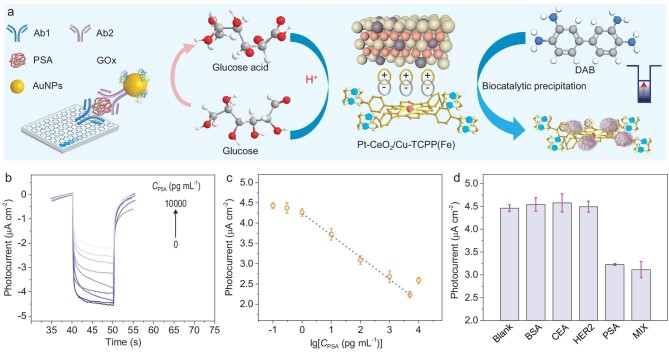
(a) Mechanism diagram of PEC immunosensor for PSA detection. (b) Photocurrent response to different concentrations of PSA (0–10 000 pg mL^−1^). (c) Logarithmic calibration curve of the PEC immunosensor within the range of 1–5000 pg mL^−1^. (d) Selectivity of the PEC immunosensor for PSA detection (*C*_PSA_ = 100 pg L^−1^, *C*_other_ = 1000 pg L^−1^). BSA: bovine serum albumin; CEA: carcinoembryonic antigen; HER2: human epidermal growth factor receptor-2; MIX: mixture.

In addition to focusing on the photoelectric response of heterojunctions, functional sensing interfaces are also important for PEC sensing. Previous studies have reported that CeO_2_ and CuTCPP(Fe) exhibit peroxidase-like activity [40,41]. The atomic Pt doping increased the peroxidase (POD)-like activity of CeO_2_ by 9.1 times under the optimal pH values (Fig. S21). Owing to the excellent POD-like activity, Pt-CeO_2_/CuTCPP(Fe) can catalyze the oxidation of 3,3'-diaminobenzidine to produce brown precipitation in the presence of H_2_O_2_, inhibiting the transfer of carriers, thus reducing the photoelectric response. Significantly, the POD-like activity of Pt-CeO_2_/CuTCPP(Fe) exhibits an obvious pH-dependent property (Fig. S22), which in turn influences the photoelectric response (Fig. S23). Thus, based on the glucose oxidase (GOx)-regulated pH via oxidizing glucose to produce glucose acid, ELISA monitoring of PSA was achieved (Fig. 5a). Then, the optimum incubation time for PSA antigen-antibody immunoreaction was revealed (Fig. S24) and the detection feasibility was verified (Fig. S25a). EIS also displays the feasibility of the detection of PSA (Fig. S25b). By virtue of the steric hindrance effect, the photoelectric behavior of heterojunction is gradually inhibited with the increase in PSA concentration (Fig. 5b). The photocurrent is linearly correlated with the logarithm of PSA within the scope of 1–5000 pg mL^−1^ (Fig. 5c). The linear fitting equation is *I* (nA) = 4.25–0.544lg*C*_PSA_ (*R*^2^ = 0.998), and the limit of detection (S/N = 3) is 0.71 pg mL^−1^. Compared with currently reported methodologies (Table S2), the PEC immunosensor in this study exhibits satisfactory sensitivity. The results in Fig. 5d demonstrate the excellent selectivity of the prepared sensor. In addition, the proposed PEC immunosensor manifests outstanding stability and reproducibility (Fig. S26). PSA detection was conducted using human serum samples to assess the potential application of Pt-CeO_2_/CuTCPP(Fe) *p-n* junction-based PEC immunosensor. As manifested in Table S3, the investigation results of immunosensors are approximate to those of the chemiluminescence method in the hospital, with the relative standard deviation in the range of 1.2%–2.6%. The results above indicate the wide-ranging potential applications of the proposed PEC immunosensor in clinical diagnosis and biomedical research.

## CONCLUSION

In this study, we present an innovative methodology for the deliberate regulation of chemically bonded interfaces in heterojunctions to accelerate charge separation through atomic-level donor doping. Our findings demonstrate that the incorporation of atomic-level dopant impurities allows for the optimization of the interface barrier and the built-in electric field of the *p-n* junction, thereby providing a substantial driving force for charge transfer. Moreover, the introduction of Pt atoms facilitates the transformation from Fe–O_A_–Ce to Fe–O_L_ electron bridges, resulting in a significant reduction in the carrier transport distance. As anticipated, this modification leads to a 2.5-fold enhancement in the photoelectric response of the *p-n* junction. By coupling this advancement with the exceptional biocatalytic activity of CuTCPP(Fe) and Pt-CeO_2_, we successfully achieve sensitive monitoring of PSA. Overall, our work presents promising prospects for precisely modulating interfacial charge separation and transfer within heterojunctions.

## Supplementary Material

nwae465_Supplemental_File

## Data Availability

All data supporting the findings of this study are included within the article and its Supplementary Information, as well as from the corresponding author upon reasonable request.
