# Supplementary material for: Lattice atom-bridged chemical bond interface facilitates charge transfer for boosted photoelectric response
Source: Natl Sci Rev. 2024 Dec 26;12(3):nwae465. doi: 10.1093/nsr/nwae465 (PMC11804805; doi:10.1093/nsr/nwae465)
Supplement: nwae465_Supplemental_File [file nwae465_supplemental_file.pdf]

Supplementary Information

Lattice Atom-Bridged Chemical Bond Interface Facilitates Charge Transfer for Boosted Photoelectric Response

Mingwang Liu,[1] Wenhong Yang,[1] Runshi Xiao,[1,2] Jinli Li,[1] Rong Tan,[1] Ying Qin,[1] Yuxuan Bai,[1] Lirong Zheng,[3] Liuyong Hu,[2] Wenling Gu,[1] and Chengzhou Zhu,[1*]

[1] State Key Laboratory of Green Pesticide, International Joint Research Center for Intelligent Biosensing Technology and Health, College of Chemistry, Central China Normal University, Wuhan 430079 (P. R. China)

[2] Hubei Key Laboratory of Plasma Chemistry and Advanced Materials, Hubei Engineering Technology Research Center of Optoelectronic and New Energy Materials, Wuhan Institute of Technology, Wuhan 430205 (P. R. China)

[3] Institute of High Energy Physics, Chinese Academy of Sciences, Beijing 100049 (P. R. China)

*Corresponding author.
E-mail: czzhu@ccnu.edu.cn (Chengzhou Zhu)

1. Experimental Procedures

Chemical and Materials

Hydrogen peroxide (H_2O_2), sodium borohydride ($NaBH_4$), sodium hydroxide (NaOH), acetic acid (HAc), dipotassium phosphate (KH_2PO_4), monopotassium phosphate trihydrate ($K_2HPO_4 \cdot 3H_2O$) and sodium acetate (CH_3COONa) were supplied by Sinopharm Chemical Reagent Co., Let (Shanghai, China). Bovine serum albumin (BSA) was obtained from Sigma-Aldrich (USA). Prostate-specific antigen (PSA) and PSA antibodies (coating and labeling) were bought from Shanghai Linc-Bio Science Co. Ltd (Shanghai, China). 3,3'-diaminobenzidine tetrahydrochloride (DAB), Glucose (Glu), glucose oxidase (GOx), K_2PtCl_4 and 3,3',5,5'-Tetramethylbenzidine (TMB) were purchased from Shanghai Aladdin Bio-Chem Technology Co., Ltd. Trifluoroacetic acid was from Shanghai Yuanye Biotechnology Co., Ltd. The carcinoembryonic antigen (CEA) and human epidermal growth factor receptor-2 (HER2) were purchased from Sangon Biotech (Shanghai) Co., Ltd. $Cu(NO_3)_2 \cdot 3H_2O$, $Ce(NO_3)_3 \cdot 6H_2O$, 3-Hydroxytyramine hydrochloride (DA) and polyvinyl pyrrolidone (PVP) were from Macklin Reagent Co., Ltd. Fe(III) tetra(4-carboxyphenyl)porphine chloride TCPP(Fe) and tetrakis(4-carboxyphenyl)porphyrin (TCPP) were supplied by Leyan. The 96-well plates were purchased from Thermo Fisher Scientific Inc. Indium tin oxide glass (ITO slices, square resistance 10 Ω, thickness 1.1 mm) was ordered from Foshan Yuanjingmei Glass Co., Ltd. Other chemicals were analytical reagents, and all solutions used in the experiment were prepared with pure water produced by the water purification system (Milli-Q water system, Millipore) with a resistivity of >18.2 MΩ cm.

Apparatus

Scanning electron microscope (SEM) images were obtained by a Quanta JEM-6700F (FEI, United States). Transmission electron microscopy (TEM) images, high-resolution transmission electron microscopy (HRTEM) images and high-angle annular dark-field scanning transmission electron microscopy (HAADF-STEM) were characterized using a Tecnai G2 F30 (FEI, United States). X-ray powder diffraction (XRD) characterization was carried out by a D8 ADVANCE (Bruker, Germany). Zeta potential measurements were performed by Zetasizer Nano ZS (Malvern Instruments). Variable-temperature electron paramagnetic resonance (EPR) spectra were obtained at 77K using the Bruker A300 (Germany). The 3D surface potential was measured by Dimension Icon (Bruker, Germany) atomic force microscope. X-ray photoelectron spectroscopy (XPS) measurements were performed by a VG Multilab 2000 (Thermo Fisher, United States). An LED lamp (λ = 365 nm) was used as the light source to examine the excited XPS spectra. All XPS spectra were calibrated with reference to the C1 peak of 284.8 eV before analysis. The X-ray absorption fine structure spectra (Pt L_3-edge and Fe K-edge) were collected at 1W1B station in Beijing Synchrotron Radiation Facility (BSRF). The element contents were obtained by inductively coupled plasma optical emission spectrometry (ICP-OES) (Agilent 8800). The ultraviolet-visible (UV-Vis) diffuse-reflectance absorption spectra were measured on a UV-Vis spectrophotometer (Agilent Technologies, Cary 100) using $BaSO_4$ as reflectance standard reference. Photoluminescence (PL) spectra were recorded at room temperature on a fluorescence spectrophotometer (Edinburgh Instruments, FLS1000) under the excitation of a 350 nm light source. Electrochemical impedance spectroscopy (EIS) and Mott-Schottky (MS) measurements were measured on a CHI 660E electrochemical workstation (Shanghai Chenhua Apparatus Corporation, China) with a three-electrode system, in which 5 mM $K_3[Fe(CN)_6]/K_4[Fe(CN)_6]$ (1:1) containing 0.1 M KCl was used as the electrolyte of EIS in a frequency range of 0.1 Hz-100 kHz, and 0.2 M Na_2SO_4 aqueous solution was adopted as the electrolyte of MS. The photoelectrochemical test was performed on a system containing a CHI842d electrochemical workstation (Shanghai Chenhua Instrument Co., Ltd.) with a three-electrode system, where a modified ITO electrode (coating area = 0.071 cm^{-2}), a Pt wire, and a saturated Ag/AgCl electrode were served as working electrode (WE), counter electrode (CE), and reference electrode (RE), respectively. 500 W Analog Daylight Xenon Light

Source (PLS-FX300HU, Beijing Perfect Light Technology Co., Ltd.) was used as exciting light in this work. The absorbance spectra were obtained from a multimode reader (Tecan Spark, Switzerland).

Synthesis of cerium dioxide (CeO_2) nanoparticle.

CeO_2 nanoparticles were prepared with a reported method with some modifications. [1] 10 mmol of cerium nitrate hexahydrate ($Ce(NO_3)_3·6H_2O$) and 320 mL of 0.39 M NaOH were added to a 1000 mL beaker. The mixture was stirred at 1000 rpm for 30 h at room temperature. The products obtained are collected and washed with ethanol and deionized water three times by centrifugation at 10000 rpm for 10 min, then dried under 60 °C for 8 hours.

Synthesis of Pt-doped CeO_2 (Pt-CeO_2) nanoparticle.

For the synthesis of Pt-CeO_2 [2], 100 mg CeO_2 nanoparticles were added into 8 mL of the aqueous solution of PVP (16 mg) and K_2PtCl_4, the mass of Pt accounted for 1%, 5% and 10% of CeO_2, respectively. The mixture was heated at 95 °C for 20 min. At the same time, 1 mL of fresh $NaBH_4$ solution (75 mM) was prepared and added to the above solution dropwise. After being maintained at 95 °C for 30 min, the resulting solution was cooled and washed with water several times.

Synthesis of Pt-CeO_2/CuTCPP(Fe) p-n junction.

CuTCPP(Fe) was prepared with a reported method with some modifications. [3] First, 12 mg of $Cu(NO_3)_2·3H_2O$ (0.05 mmol), 50 μL of trifluoroacetic acid (1.0 M) and 50.0 mg of PVP were dissolved in 60 mL of the mixture of DMF and ethanol (V: V = 3:1) in a 250 mL round bottom flask. Then, the obtained solution was mixed with 22 mg of TCPP(Fe) (0.025 mmol) dissolved in 20 mL of the mixture of DMF and ethanol (V: V = 3:1). After the solution was sonicated for 15 min, the round bottom flask was heated to 80 °C and kept for 3 h. The resulting dark brown products were washed twice with ethanol and collected by centrifuging at 11000 r.p.m. for 15 min. The obtained CuTCPP(Fe) nanosheets were redispersed in pure water. The p-n junction was prepared by electrostatic interaction, CuTCPP(Fe) and Pt-CeO_2 were mixed, ultrasonic for 10 min, and then stirred overnight.

Peroxidase-like (POD-like) Activity Assay.

5 μL of 0.5 mg mL^{-1} of CuTCPP(Fe) was added to 200 μL of an acetate buffer solution with different pH values (pH range from 3 to 7), to which 50 μL H_2O_2 (10 mM) and 50 μL TMB (1 mM) were added. After being kept at room temperature for 3 minutes, the resulting mixed solution was carried out by UV-Vis scanning by a multimode reader. Similarly, 5 μL of 0.5 mg mL^{-1} of CeO_2 or Pt-CeO_2 was added to 200 μL of an acetate buffer solution with different pH values (pH range from 3 to 9), to which 50 μL H_2O_2 (10 mM) and 50 μL TMB (1 mM) were added. 5 μL of 1 mg mL^{-1} of Pt-CeO_2/CuTCPP(Fe) was added to 200 μL of an acetate buffer solution with different pH values (pH range from 3 to 7), to which 50 μL H_2O_2 (10 mM) and 50 μL TMB (1 mM) were added.

Synthesis of Au nanoparticles (Au NPs).

In brief, 3 mL of 1 wt% sodium citrate solution was added to 200 mL of boiling 0.01 wt% $HAuCl_4$ solution, and the mixed solution was refluxed for 1h.

Preparation of GOx-AuNPs-Ab_2. [4]

Firstly, Au NPs (40.0 mL, 5.0 ng mL^{-1}) were adjusted to pH 9.5 by using 0.1 M Na_2CO_3 aqueous solution. Then, 1.6 mL of GOx (0.5 mg mL^{-1}) and 400 μL of Ab_2 (0.5 mg mL^{-1}) were injected into Au NPs and gently shaken for 60 min at room temperature on a shaker. After that, 800 μL of polyethylene glycol (1.0 wt %) was added into the suspension and the mixture was further incubated for 12 h at 4 ℃. Finally, GOx-AuNPs-Ab_2 conjugates were obtained by centrifugation at 4 ℃ (10 min, 14 000 r.p.m.), and dispersed in 8.0 mL of 2.0 mM sodium carbonate solution containing 1.0 wt % BSA, pH 7.4, and stored at 4 ℃ for further use.

PEC immunization test protocol

Before the preparation of photoelectrodes, the indium tin oxide (ITO) slices (0.5 × 2 cm) were thoroughly washed with ultrapure water and ethanol under ultrasonication and dried at 60 °C. Next, 10 μL of

Pt-CeO_2/CuTCPP(Fe) (1 mg mL^{-1}) was evenly deposited on an ITO electrode with an actual coating area of 0.071 cm^{-2} and baked at 50 ℃. The obtained electrodes were employed as the working electrode for the subsequent photoelectrochemical detection. The immunoassay is described below. [5] 50 μL of anti-PSA monoclonal antibody (Ab_1, 10 μg mL^{-1}) was dropped into a 96-well microplate containing sodium carbonate buffer (0.05 M, pH 9.6) and then incubated at 4 ℃ for 24 h. After washing three times with PBS buffer (10 mM, containing 0.05% Tween 20, pH 7.4), the prepared Ab_1-coated microplates were covered with 100 μL blocking buffer (10 mM PBS, containing 1.0 % BSA, pH 7.4) at 37 ℃ for 1 h. Then, different concentrations of PSA standard solution (50 μL) were added to Ab_1-coated microplates and gentle shaking at 37 ℃ for 1 h. Subsequently, the as-prepared GOx-AuNP-Ab_2 (50 μL) was introduced to perform a sandwich-type immunoreaction and incubated at 37 ℃ for 1 h. The non-specific conjugations were removed by washing with PBS. Finally, 150 μL of glucose solution (10 mM) was added to the incubation cell and reacted at 37 ℃ for 60 min. During this process, glucose acid was produced to participate in subsequent reactions. DAB (2 mM, 50 μL) and H_2O_2 (100 mM, 50 μL) were added to the mixed solution. 10 μL of the above-mixed solutions were dropped on the Pt-CeO_2/CuTCPP(Fe) electrodes, and then the electrodes were incubated at 37 ℃ for 45 minutes. After the reaction, the electrodes were immersed in deionized water, taken out immediately, and air-dried naturally. Then, the photoelectric performance test was carried out. Photocurrent measurement was carried out using a CHI 842D electrochemical workstation in a standard three-electrode configuration with the photoelectrode as the WE, a platinum wire as the CE, and an Ag/AgCl (saturated with KCl) electrode as the RE, respectively. 500 W Analog Daylight Xenon Light Source (PLS-FX300HU, Beijing Perfectlight Technology Co., Ltd.) was used as exciting light in this work. The light intensity was adjusted to 100 mW cm^{-2}. All PEC measurements were performed in phosphate buffer solution (PBS, 0.1 M, pH 7.4) at a bias of -0.2 V. R_{ct} was obtained by electrochemical impedance spectroscopy (EIS) under the same reaction conditions. EIS was measured on a CHI 660E EC workstation (Shanghai Chenhua Apparatus Corporation, China) with a three-electrode system in 1 M KCl solution containing 5 mM $K_3[Fe(CN)_6]/K_4[Fe(CN)_6]$ (1:1) mixture as the electrolyte in a frequency range of 0.1 Hz-100 kHz. All the PEC tests were obtained at room temperature.

Bandgap calculation

The bandgap energy (E_g) can be estimated from the following formula [6]:

$(\alpha h\nu)^{1/n} = A (h\nu - Eg)$

Where α, h, ν, and A represent the absorbance coefficient, Planck's constant, light frequency, and proportionality constant, respectively. The value of n depends on the type of semiconductors (n=1/2 for direct-gap semiconductors and n=2 for indirect-gap semiconductors), for CeO_2 and CuTCPP(Fe), n=1/2.

Theoretical calculations method

Our spin-polarized density functional theory (DFT) calculations[7,8] were carried out in the Vienna ab initio simulation package (VASP) based on the plane-wave basis sets with the projector augmented-wave method. [9] The exchange-correlation potential was treated by using a generalized gradient approximation (GGA) with the Perdew-Burke-Ernzerhof (PBE) parametrization. [10] To overcome the deficiency of GGA, the GGA + U method was adopted in our calculations. The value of the effective Hubbard U was set as 5.0 eV for Ce, according to the previous study. [11] The van der Waals correction of Grimme's DFT-D3 model was also adopted.[12]

A supercell of CeO_2 with {111} surface exposed was constructed containing 3 × 5 × 1 unit orthorhombic cells. The {111} surface was cleaved from the optimized CeO_2 primitive cell. A vacuum region of more than 12 Å was applied to avoid the interaction between adjacent images. The {111} slab model was transformed from a hexagonal to orthorhombic cell before making the supercell. The TCPP(Fe) molecular cluster was used to simulate the MOF structure. The energy cutoff was set to be 400 eV. The Brillouin-zone integration was sampled with a Γ point (1 × 1 × 1).[13] The structures were fully relaxed until the maximum force on each atom

was less than 0.04 eV/Å, and the energy convergent standard was 10^{-5} eV. The different charge density can be defined as $\Delta\rho = \rho_{\mathrm{AB}} - \rho_{\mathrm{A}} - \rho_{\mathrm{B}}$, where ρ_{AB}, ρ_{A}, and ρ_{B} are the electron densities of the combined structure and the separated ones.

2. Figures S1-S26.

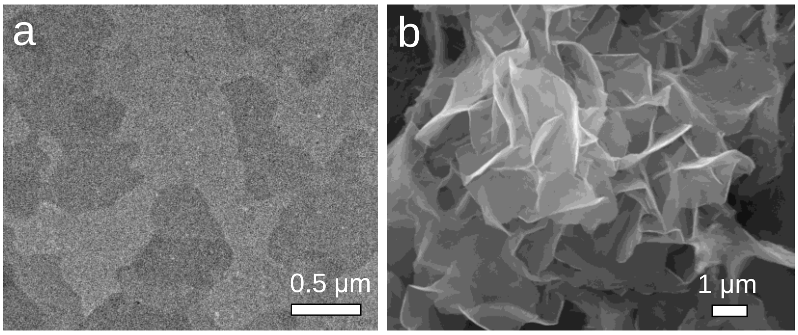

Figure S1. (a) TEM and (b) SEM images of CuTCPP(Fe).

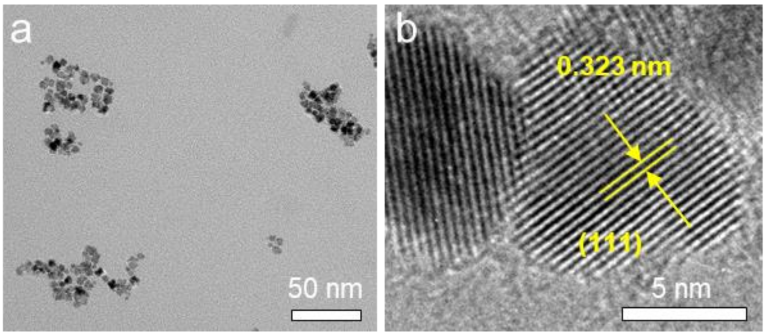

Figure S2. (a) TEM and (b) HRTEM images of CeO_2.

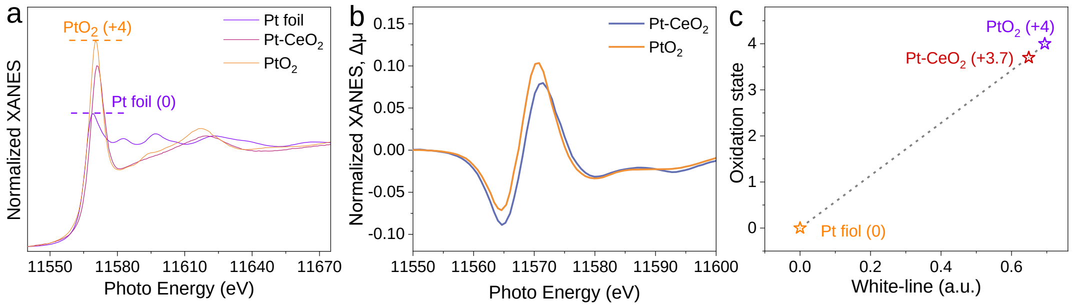

Figure S3. (a) Normalized Pt L_3-edge XANES spectra. (b)The normalized ΔXANES spectra for Pt L_3-edge using Pt foil as the reference. The oxidation states are fitted by integrating the area of the white-line peak from 11564 to 11580eV. (c) The oxidation states of Pt-CeO_2 samples.

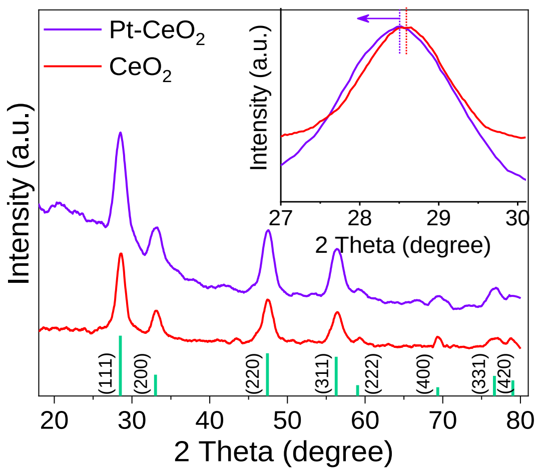

Figure S4. XRD patterns of Pt-CeO_2 and CeO_2 indexed to JCDPS no. 34-0394.

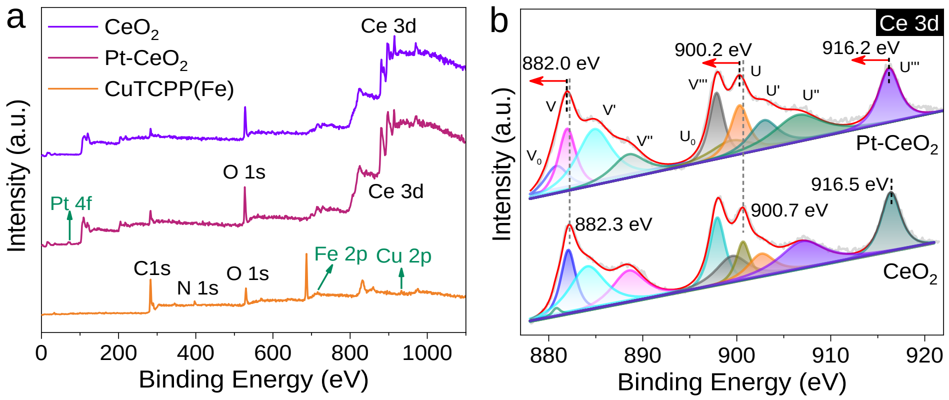

Figure S5. (a) XPS spectra of CeO_2, Pt-CeO_2 and CuTCPP(Fe). (b) High-resolution Ce 3d XPS spectra of Pt-CeO_2 and PtO_2.

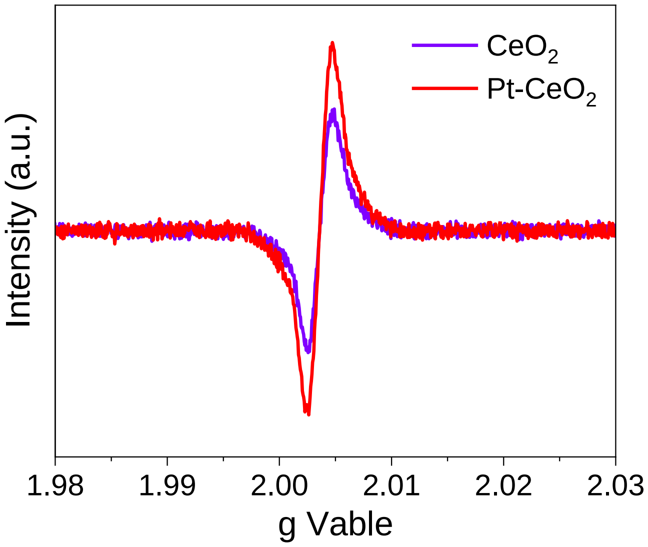

Figure S6. Variable-temperature electron paramagnetic resonance spectra of CeO_2 and Pt-CeO_2.

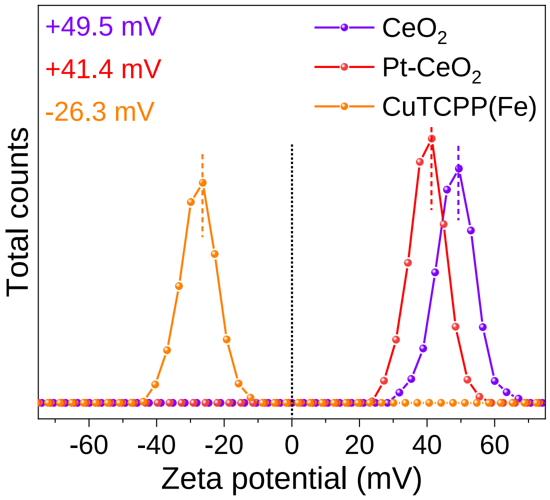

Figure S7. Zeta potential of the CeO_2, Pt-CeO_2 and CuTCPP(Fe) dispersed in pure water.

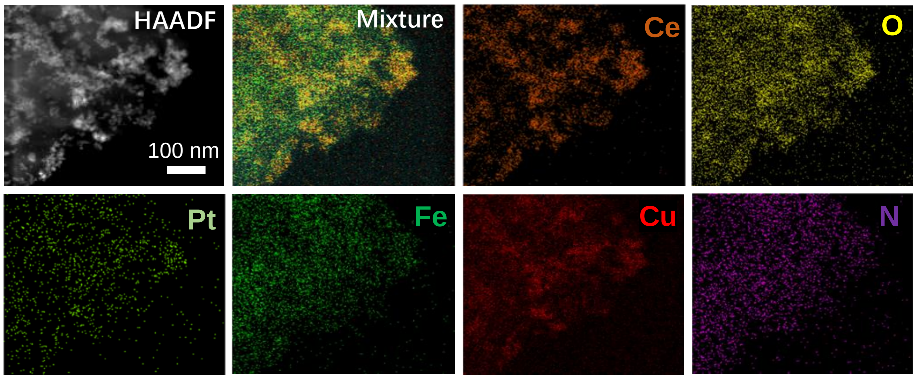

Figure S8. High-angle annular dark-field scanning TEM (HAADF-STEM) image and the corresponding energy-dispersive X-ray spectroscopy (EDS) mapping images of Pt-CeO_2/CuTCPP(Fe).

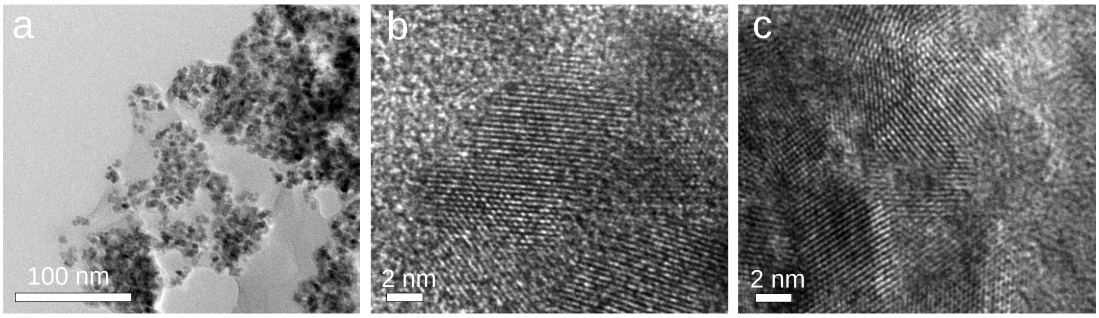

Figure S9. (a) TEM and (b) HRTEM images of CeO_2/CuTCPP(Fe). (c) HRTEM image of Pt-CeO_2/CuTCPP(Fe).

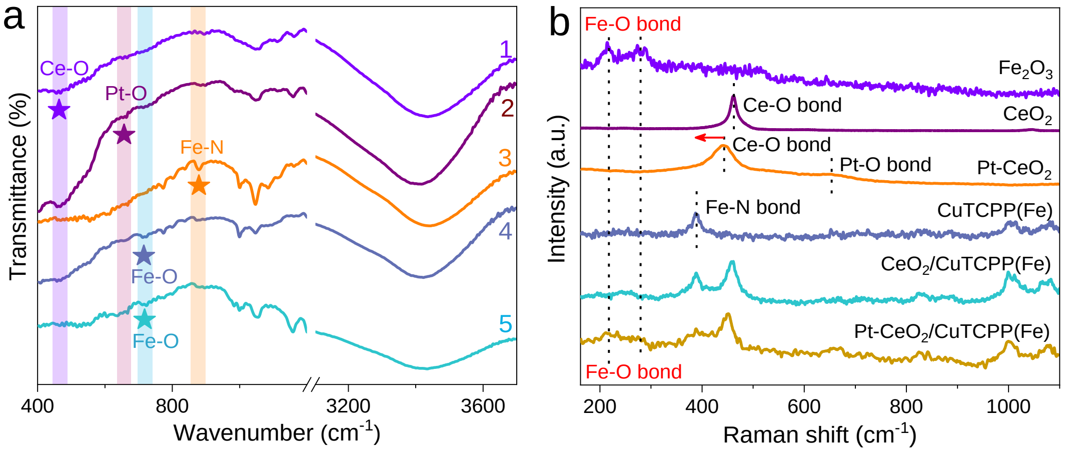

Figure S10. (a) FTIR spectroscopy of 1(CeO_2), 2(Pt-CeO_2), 3(CuTCPP(Fe)), 4(CeO_2/CuTCPP(Fe)) and 5(Pt-CeO_2/CuTCPP(Fe)). (b) Raman spectra of Fe_2O_3, CeO_2, Pt-CeO_2, CuTCPP(Fe), CeO_2/CuTCPP(Fe) and Pt-CeO_2/CuTCPP(Fe).

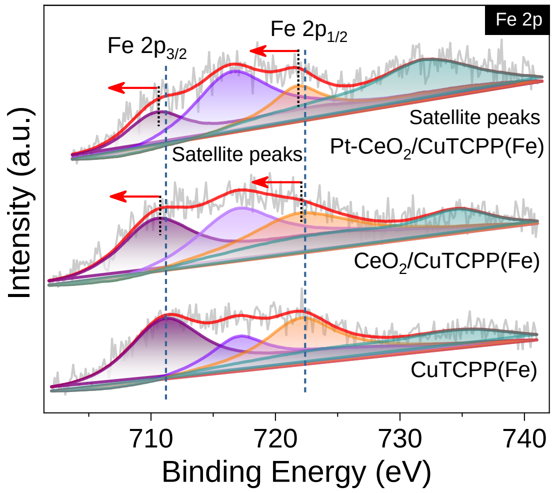

Figure S11. High-resolution Fe 2p XPS spectra of CuTCPP(Fe), CeO_2/CuTCPP(Fe) and Pt-CeO_2/CuTCPP(Fe).

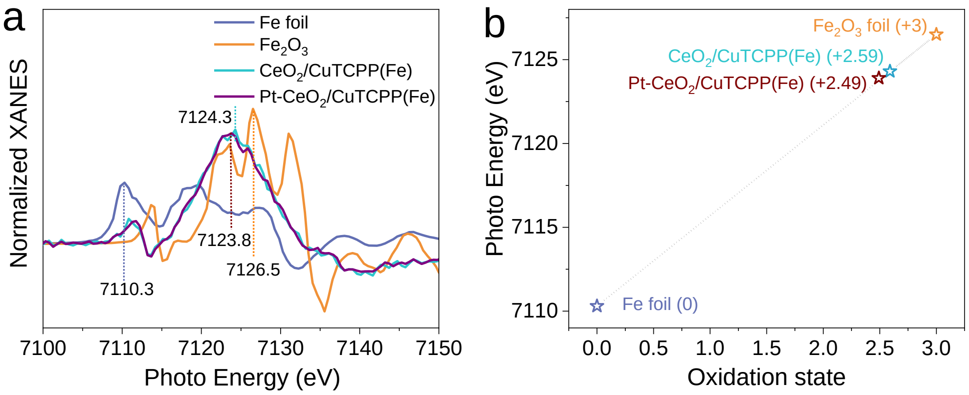

Figure S12. (a) First-derivative XANES curves of CeO_2/CuTCPP(Fe) and Pt-CeO_2/CuTCPP(Fe) and the references at Fe K-edge. (b) The average oxidation states of Fe from the XANES spectrum.

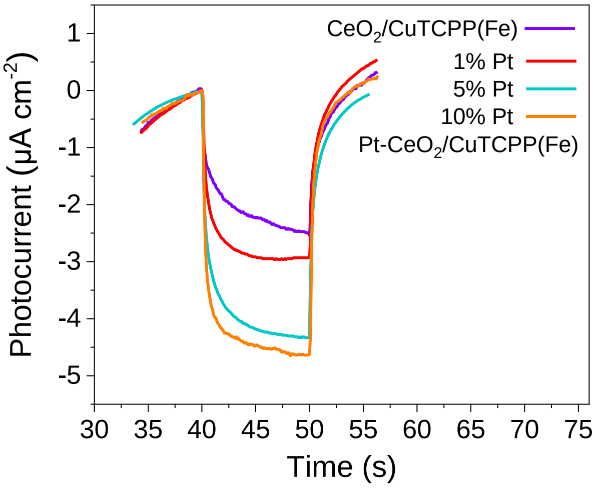

Figure S13. Photocurrent response of heterojunction doped with the different Pt contents.

.

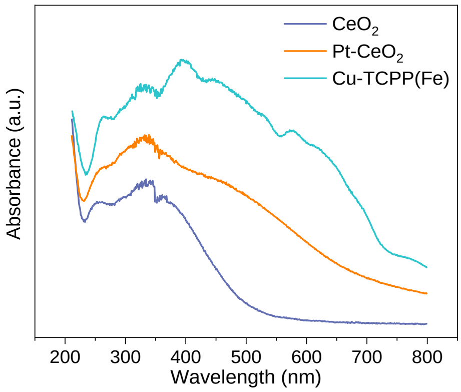

Figure S14. (a) UV−Vis diffuse reflectance spectra of CeO_2, Pt-CeO_2 and CuTCPP(Fe).

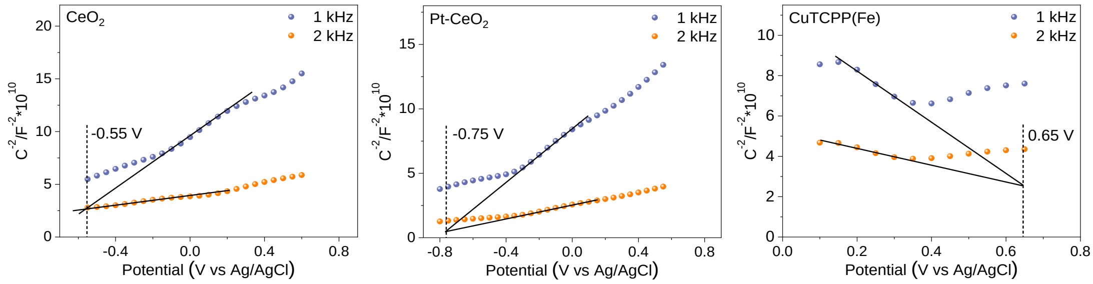

Figure S15. Mott-Schottky measurements of CeO_2, Pt-CeO_2 and CuTCPP(Fe).

The positive slopes of the Mott-Schottky curve tangent of CeO_2 and Pt-CeO_2 indicate their *n*-type characteristics.[7] CuTCPP(Fe) shows *p*-type characteristics. The flat-band (E_{fb}) potential of CeO_2, Pt-CeO_2 and CuTCPP(Fe) were estimated as -0.55 V, -0.75 V and 0.65 V, respectively (vs Ag/AgCl, pH 7). The potential was measured against an Ag/AgCl reference and converted to NHE potentials by using E(NHE) = E(Ag/AgCl) + 0.197 V. In general, The E_{fb} potential position of p-type semiconductor is above the VB (higher than about 0.1 V), and E_{fb} potential position of n-type semiconductor is below the CB (lower than about 0.1 V). Therefore, the CB of CeO_2 and Pt-CeO_2 are -0.65 V and -0.85 V, respectively (vs NHE, pH 7). The VB of CuTCPP(Fe) is 0.75 V (vs NHE, pH 7). Combined with E_g values, the VB of CeO_2 and Pt-CeO_2 can be calculated to be 2.05 and 1.15 V, respectively (vs. NHE, pH 7). The CB of CuTCPP(Fe) is -0.92 V.

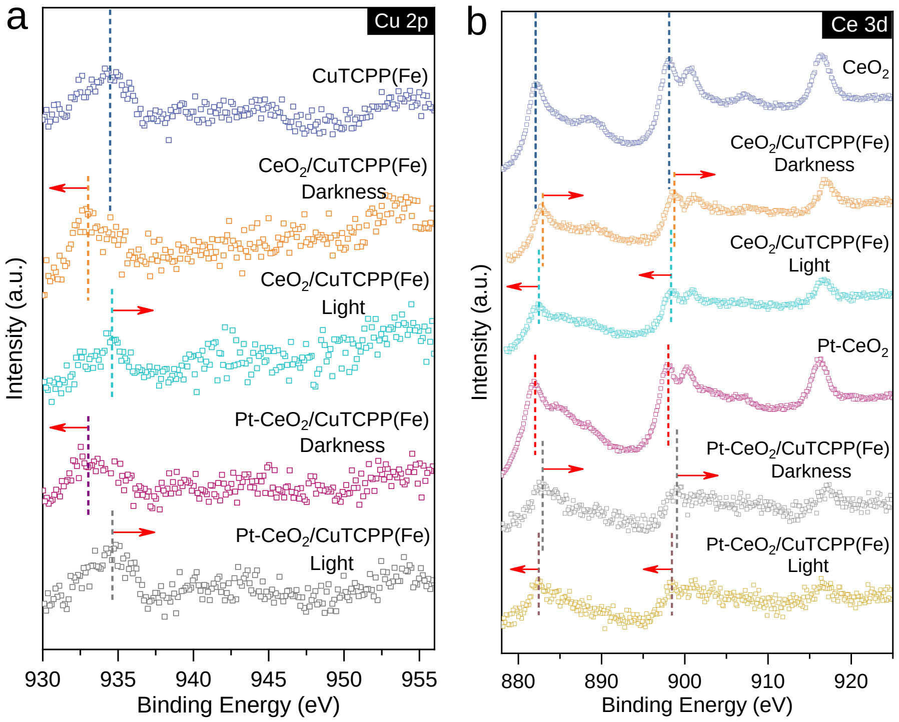

Figure S16. High-resolution (a) Cu 2p and (b) Ce 3d XPS spectra tested in darkness and under illumination (λ = 365 nm).

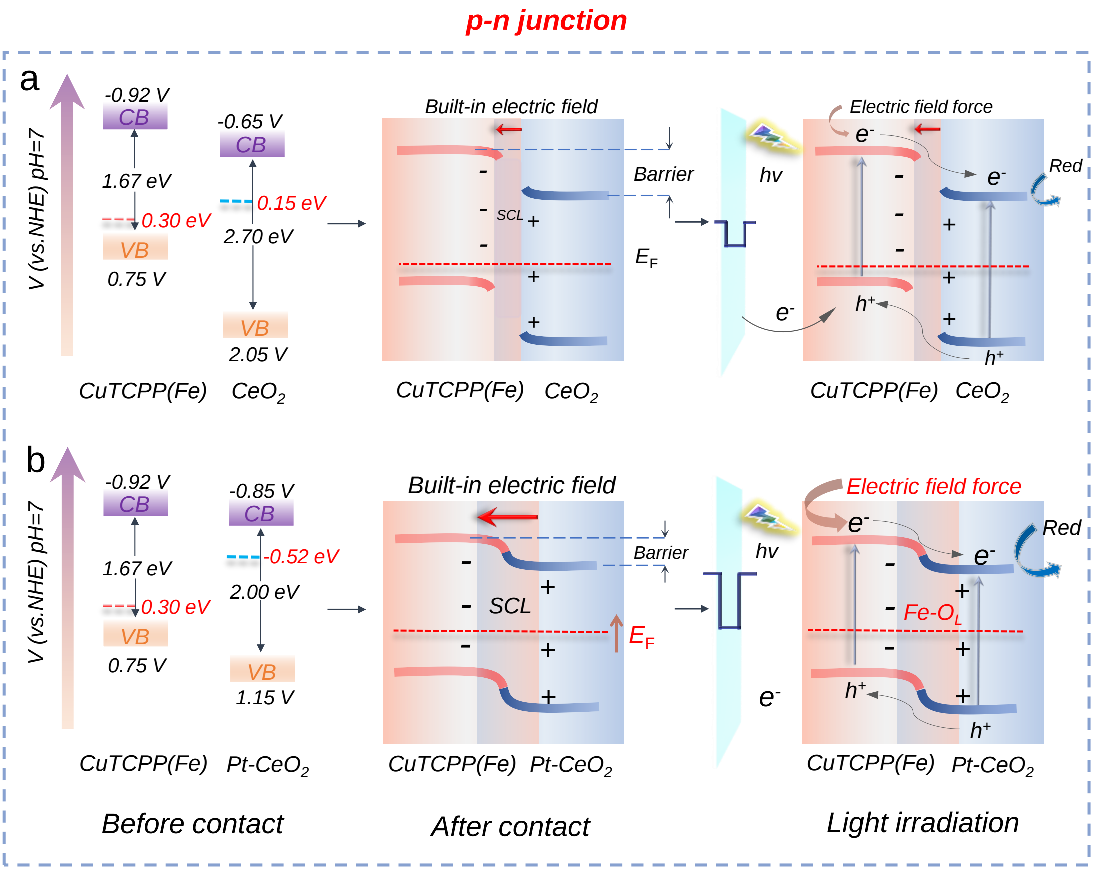

Figure S17. Schematic diagram of charge transfer path of (a) CeO_2/CuTCPP(Fe) and (b) Pt-CeO_2/CuTCPP(Fe) p-n junction.

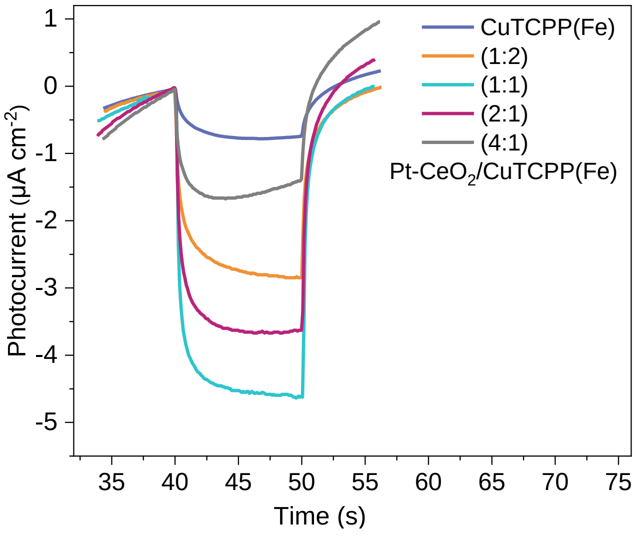

Figure S18. Photocurrent response of p-n junctions with different mass proportions.

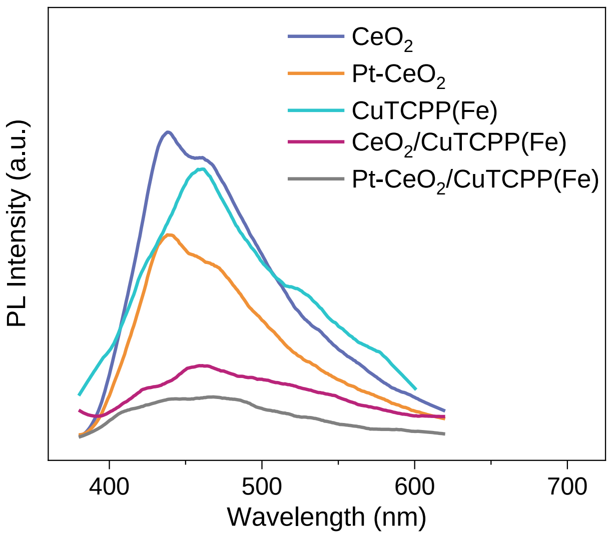

Figure S19. Steady-state PL spectra of photoelectrode.

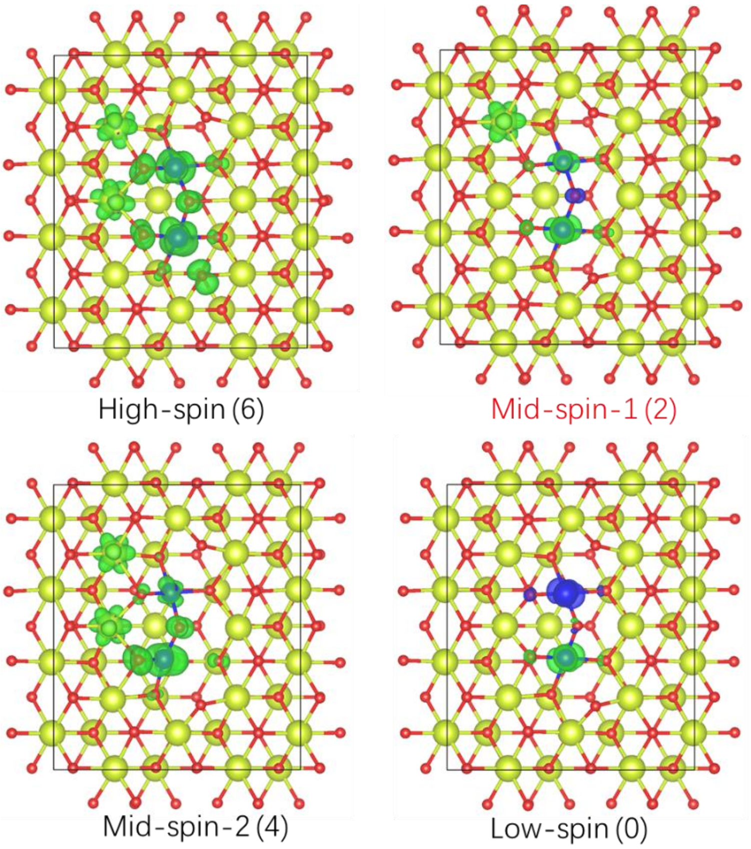

Figure S20. Structural model optimization of Pt-CeO_2.

Magnetic moment tests were conducted on four different Pt-CeO_2 structures to investigate the behavior of the model under high, medium, and low spin conditions. The net spin values of the model are shown in parentheses, while density surfaces indicate the positions of single-electron distribution. Green represents an upward spin orientation, while blue represents a downward spin orientation. Among the aforementioned four structures, the Mid-spin-1 model exhibits the lowest energy and demonstrated the presence of suitable Pt-O-Pt and Ce-O-Ce sites for oxygen binding with iron.

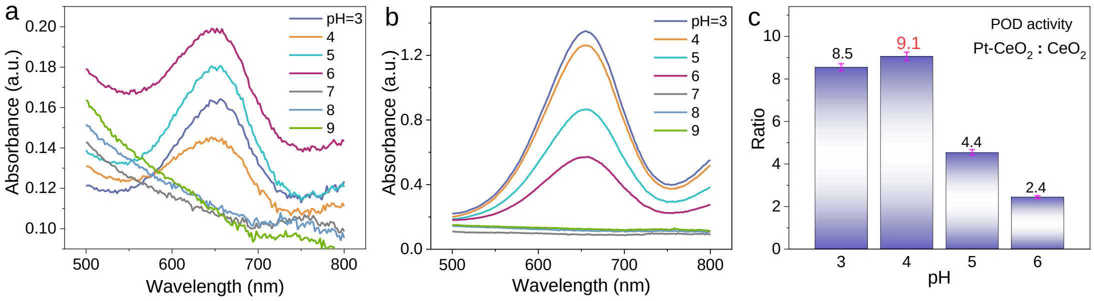

Figure S21. UV-Vis absorption of (a) CeO_2 and (b) Pt-CeO_2 at different pH values including 10 mM H_2O_2 and 1 mM TMB. (c) POD-like activity ratio of Pt-CeO_2 and CeO_2 at different pH values.

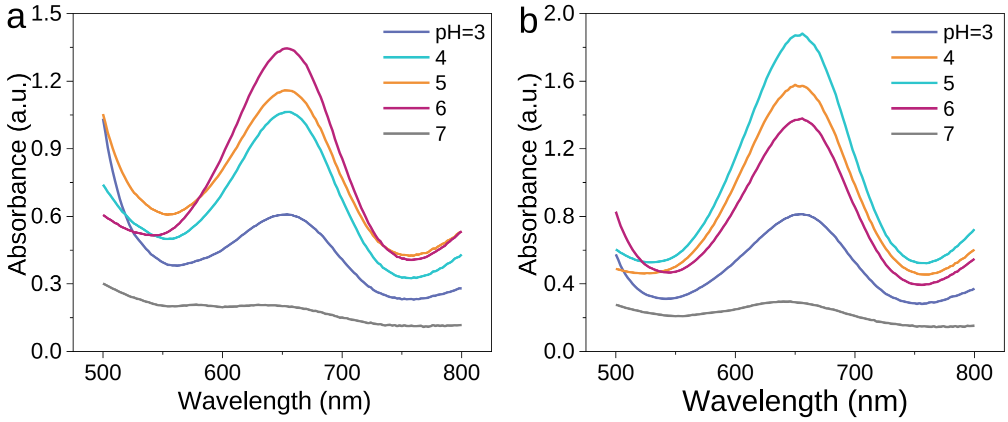

Figure S22. UV-Vis absorption of (a) CuTCPP(Fe) and (b) Pt-CeO_2/CuTCPP(Fe) at different pH values including 10 mM H_2O_2 and 1 mM TMB.

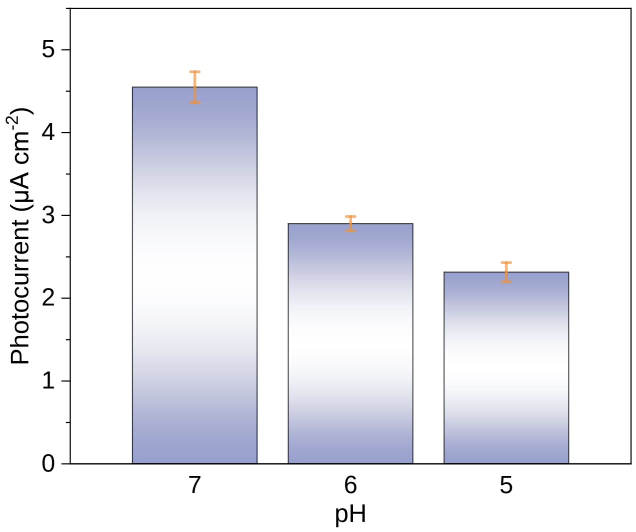

Figure S23. Photocurrent responses of Pt-CeO_2/CuTCPP(Fe) in buffer solution with different pH values containing 2 mM DAB.

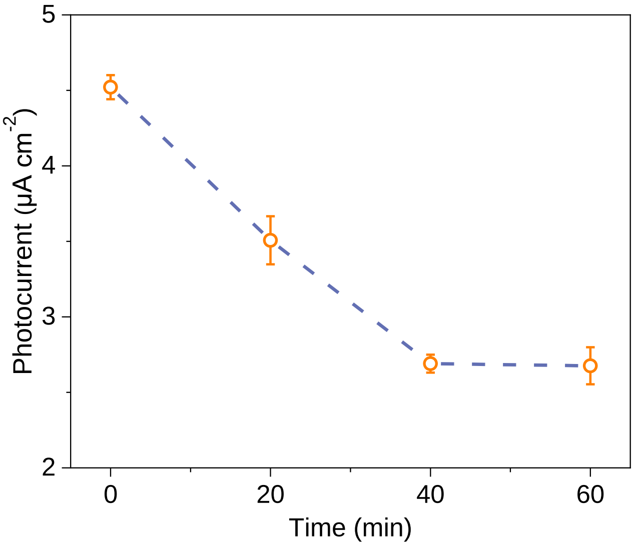

Figure S24. The incubation time for PSA (100 pg mL^{-1}) antigen-antibody immunoreaction on the photocurrents of the PEC sensor.

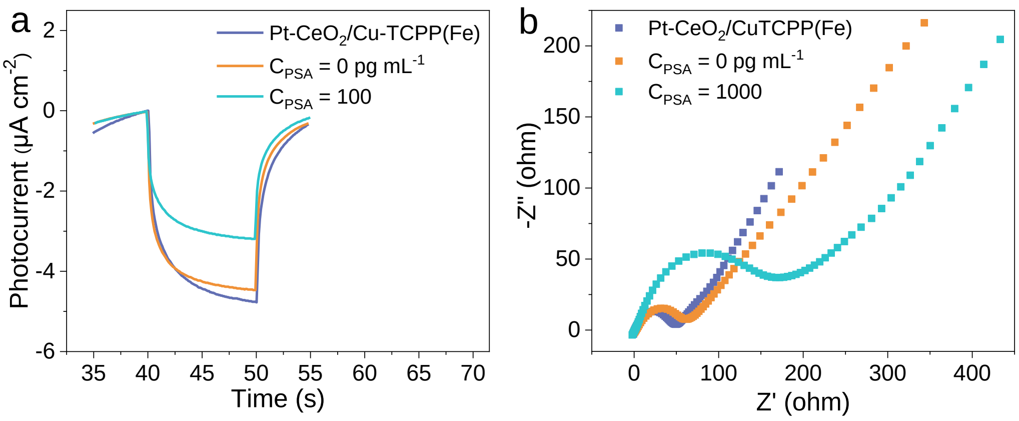

Figure S25. Photocurrent response of Pt-CeO_2/CuTCPP(Fe) and Pt-CeO_2/CuTCPP(Fe) photoelectrodes incubated at various concentrations of PSA (0 and 100 pg mL^{-1}). (b) EIS Nyquist plots of the Pt-CeO_2/CuTCPP(Fe) and Pt-CeO_2/CuTCPP(Fe) with various concentrations of PSA (0 and 1000 pg mL^{-1}) in 1 M KCl solution containing 5 mM $[Fe(CN)_6]^{3-/4-}$.

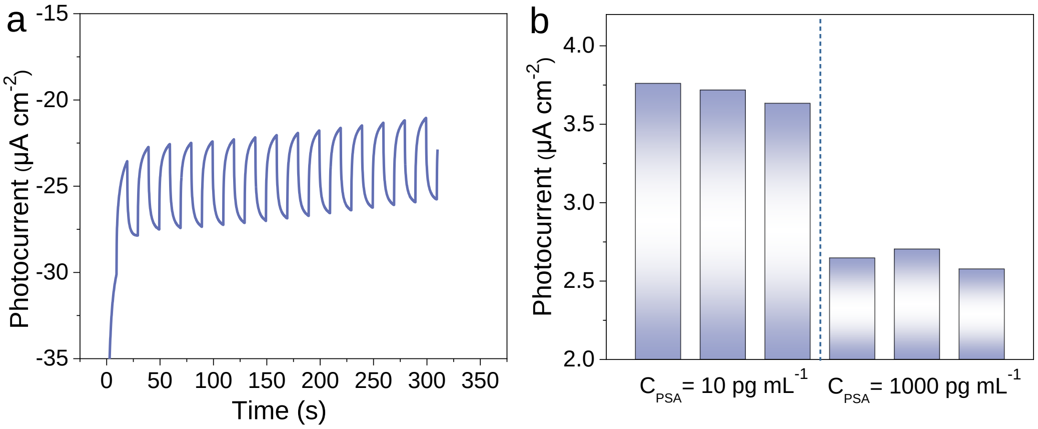

Figure S26. Time-based photocurrent responses of the PEC immunosensing system (C_{PSA} = 1 pg mL^{-1}). Repeatability of a PEC immunosensor (C_{PSA} = 10 and 1000 pg mL^{-1}).

3. Tables S1-S3.

Table S1. Structural parameters of the Fe K-edge EXAFS fitting (S_0^2=0.950).

Sample	Shell	N[a]	R (Å)[b]	σ^2 (Å^2·10^{-3}) [c]	ΔE_0 (eV)[d]	R factor (%)
CeO_2/CuTCPP(Fe)	Fe-N	3.85	1.98	6.17	2.56	0.009
	Fe-O	0.82	2.08	2.95	4.89	
Pt-CeO_2/CuTCPP(Fe)	Fe-N	4.12	2.03	1.28	10.02	0.012
	Fe-O	0.91	1.81	1.45	3.85	

[a]N: coordination numbers; [b]R: bond distance; [c]σ^2: Debye-Waller factors; d ΔE_0: the inner potential correction. Error bounds (accuracies) characterizing the structural parameters obtained by EXAFS spectroscopy are estimated to be as follows: N, ±20%; R, ±1%; σ^2, ±20%; and ΔE_0, ±20%. R factor: goodness of fit. S_0^2 was set as 0.950, which was obtained from the experimental EXAFS fit of reference FeO and FePc by fixing [a]N as the known crystallographic value and was fixed to all the samples.

Table S2. Comparison of different PSA sensors.

Methods	Materials	Linear range (ng mL^{-1})	LOD (pg mL^{-1})	Ref.
Fluorescence	Carbon dots	1-500000	300	14
	UCNPs	0.01-50	4.1	15
Electrochemistry	Pd@Au@Pt/COOH-rGO	0.012-85	8	16
	Ferrocene derivative	0.05-20	10	17
Colorimetric	Coordination polymers	0.05-100	21.1	18
Chemiluminescent	CuO NRs	0.1-60	50	19
	HRP-Au NPs	0.1-100	50	20
Photoelectrochemical	UCNPs@CdTe	0.01-5	4.8	21
	TiO_2-BPQDs	0.005-50	1	22
	CdS@NiS	0.01-50	2.9	23
	Pt-CeO_2/CuTCPP(Fe)	0.001-5	0.71	**This work**

Table S3. Detection of PSA in human serum samples.

Nos.	Serum samples (pg mL^{-1})	PEC sensor (pg mL^{-1})	RSD (n=3, %)
1	2004	1974	1.5
2	556	581	1.2
3	980	964	2.6

4. References

(1) M. J. Manto, P. Xie, C. Wang, *ACS Catal.* **2017**, *7* (3), 1931-1938.
(2) R. Yan, S. Sun, J. Yang, W. Long, J. Wang, X. Mu, Q. Li, W. Hao, S. Zhang, H. Liu, Y. Gao, L. Ouyang, J. Chen, S. Liu, X. D. Zhang, D. Ming, *ACS Nano* **2019**, *13* (10), 11552-11560.
(3) Y. Huang, M. Zhao, S. Han, Z. Lai, J. Yang, C. Tan, Q. Ma, Q. Lu, J. Chen, X. Zhang, Z. Zhang, B. Li, B. Chen, Y. Zong, H. Zhang, *Adv. Mater.* **2017**, *29* (32) 1700102.
(4) Q. Lin, X. Huang, L. Lu, D. Tang, *Biosens Bioelectron* **2022**, *216*, 114679.
(5) Z. Yu, H. Gong, Y. Gao, L. Li, F. Xue, Y. Zeng, M. Li, X. Liu, D. Tang, *Small* **2022**, *18* (30), e2202564.
(6) S. Cao, B. Shen, T. Tong, J. Fu, J. Yu, *Adv. Funct. Mater.* **2018**, *28* (21) 1800136.
(7) P. Hohenberg, W. Kohn, *Phys. Rev.* **1964**, *136* (3B), B864-B871.
(8) W. Kohn, L. J. Sham, *Phys. Rev.* **1965**, *140* (4A), A1133-A1138.
(9) P. E. Blochl, *Phys. Rev. B Condens Matter* **1994**, *50* (24), 17953-17979.
(10) P. P. John, B. Kieron, E. Matthias, *Phys. Rev. Lett.* **1996**, *77*, 3865-3868.
(11) M. Nolan, *J. Mater. Chem.* **2011**, *21* (25) 9160-9168.
(12) S. Grimme, J. Antony, S. Ehrlich, H. Krieg, *J. Chem. Phys.* **2010**, *132* (15), 154104.
(13) H. J. Monkhorst, J. D. Pack, *Phys. Rev. B* **1976**, *13* (12), 5188-5192.
(14) H. Miao, L. Wang, Y. Zhuo, Z. Zhou, X. Yang, *Biosens. Bioelectron.* **2016**, *86*, 83-89.
(15) Q. Han, X. Zhao, X. Zhang, N. Na, J. Ouyang, *Sensor Actuat. B-Chem.* **2022**, *360*, 131663.
(16) S. C. Barman, M. F. Hossain, H. Yoon, J. Y. Park, *Biosens. Bioelectron.* **2018**, *100*, 16-22.
(17) X. Gu, Z. She, T. Ma, S. Tian, H. B. Kraatz, *Biosens. Bioelectron.* **2018**, *102*, 610-616.
(18) S. Wu, H. Tan, C. Wang, J. Wang, S. Sheng, *ACS Appl. Mater. Interfaces* **2019**, *11*, 43031-43038.
(19) J. Li, Y. Cao, S. S. Hinman, K. S. McKeating, Y. Guan, X. Hu, Q. Cheng, Z. Yang, *Biosens. Bioelectron.* **2018**, *100*, 304-311.
(20) L. Z. Zhao, Y. Z. Fu, S. W. Ren, J. T. Cao, Y. M. Liu, *Biosens. Bioelectron.* **2021**, *171*, 112729.
(21) Z. Qiu, J. Shu, J. Liu, D. Tang, *Anal. Chem.* **2019**, *91* (2), 1260-1268.
(22) H. Shi, S. Ge, Y. Wang, C. Gao, J. Yu, *ACS Appl. Mater. Interfaces* **2019**, *11* (44), 41062-41068.
(23) L. Zhu, Z. Lv, Z. Yin, D. Tang, *Anal. Chim. Acta.* **2021**, *1149*, 338215.
